# Quality assurance of malaria case management in an urban and in sub-rural health centres in Goma, Congo

**DOI:** 10.4102/phcfm.v3i1.225

**Published:** 2011-10-07

**Authors:** Prosper M. Lutala, Claude M. Kasereka, Eric K. Kasagila, John B. Inipavudu, Suleiman I. Toranke

**Affiliations:** 1 DOCS Learning Centre and Department of Family Medicine, Université de Goma, The Democratic Republic of Congo; 2 Department of Community Health, College of Medicine, School of Public Health, University of Malawi, Malawi; 3 Ministry of Health, Central-West Zone, Lilongwe, Malawi

## Abstract

**Background:**

Every year, up to three million deaths throughout the world occur as a result of malaria, 90% of which occur in Africa. Despite training providers in malaria case management and the availability of appropriate medical suppliers, there are still weaknesses in the management chain of malaria.

**Objectives:**

Our aim was to assess the quality of malaria case management in two primary health care centres in the Goma health district. Specific objectives were the assessment of quality accuracy in the dosage, the duration of treatment, the intervals between administrations, and the routes of administration of anti-malarial medication in two health centres, as well as the subsequent comparison of those two sites.

**Method:**

A descriptive retrospective study was conducted using the malaria register's review to assess two health centres in the Goma health district. Socio-demographical and clinical data were recorded and the quality was assessed against the national guidelines. Descriptive statistics with percentages and Chi-square values were computed.

**Results:**

Under-dosage was more common in CCLK (Centre Chrétien du Lac Kivu [*Lake Kivu Christian Centre*]) with 55 patients (62.5%; 95% CI, 52% – 71.8%) patients, whilst the over-dosage was present in 64 patients (80%; 95% CI, 69.9% – 87.2%) in CASOP (Caisse de Solidarité Ouvrière et Paysanne [*Fund of Solidarity Workers and Peasants*]). The duration of treatment was shorter in CCLK in 15 patients (93.7%; 95% CI, 71.6% – 98.8%); CASOP had a high rate of inappropriate intervals between the administration of drugs in 14 patients (82.3%; 95% CI, 58.9% – 93.8%). Intravenous administration rates were high in both sites with respectively 102 patients in CASOP (62.5%; 95% CI, 54.9% – 69.6%) and 61 patients in CCLK (37.4%; 95% CI, 30.3% – 45.0%). Significant differences were found between the two sites with regard to intervals of administration (*χ*^2^ = 7.11, *p* = 0.007), duration of treatment (*χ*^2^ = 8.51, *p* = 0.003), dosage (χ^2^ = 3.91, p = 0.05). The routes of administration were used in a similar manner, however, in the two sites (χ^2^ = 0.78, p = 0.37).

**Conclusion:**

Abnormalities in dosage, in the duration of treatment, in the intervals between administration and in the routes of administration were found in both sites. Consequently we conclude that success in guidelines implementation is a complex process and cannot be based only on scientific evidence, but certain contextual factors must be considered.

## Abstrait

**Contexte de l’étude:**

Chaque année, jusqu’à trois millions de décès dus au paludisme surviennent dans le monde entier, 90% d'entre eux en Afrique. En dépit de formations des prestataires dans la gestion du paludisme et la fourniture d'intrants, certaines faiblesses sont encore présentes dans la chaîne de prise en charge du paludisme.

**Objectifs:**

Notre objectif était d’évaluer la qualité de la gestion des cas du paludisme dans les deux centres de soins primaires dans la zone de santé de Goma. Les objectifs spécifiques étaient d’évaluer la qualité de la posologie, la durée du traitement, les intervalles entre les administrations et les voies d'administration des antipaludiques dans les deux centres de santé, la comparaison ultérieure de ces deux sites.

**Méthodes:**

Étude rétrospective descriptive à l'aide d'une revue du registre du paludisme pour évaluer les deux centres de santé dans le district de santé de Goma a été réalisée. Les données sociodémographiques et cliniques ont été enregistrées et la qualité a été évaluée en fonction des directives nationales. Les statistiques descriptives avec calcul des pourcentages et du chi carré ont été calculées.

**Résultats:**

Faibles doses étaient communs dans 55 patients à CCLK (Centre Chrétien du Lac Kivu), (62.5%; IC à 95%, 52% – 71.8%), tandis que le nombre des doses élevées étaient plus dans CASOP (Caisse de Solidarité Ouvrière et Paysanne [*Fund of Solidarity Workers and Peasants*]) dans 64 patients (80.0%; IC à 95%, 69.9%–87.2%). La durée du traitement était plus courte à CCLK dans 15 patients (93.7%; IC à 95%, 71.6% – 98.8%). CASOP a également eu un taux élevé d'intervalles inappropriés entre l'administration dans 14 patients (82.3%; IC à 95%, 58,9% – 93.8%). Le taux d'administration intraveineuse était élevé dans les deux sites avec respectivement 102 patients à CASOP (62.5%; IC à 95%, 54.9% – 69.6%) et 61 patients à CCLK (37.4%; IC à 95%, 30.3% – 45%). On a trouvé des différences significatives entre les deux sites en ce qui concerne les intervalles d'administration (*χ*^2^ = 7.11, *p* = 0.007), la durée du traitement (*χ*^2^ = 8.51, *p* = 0.003), la posologie (*χ*^2^ = 3.91, *p* = 0.05). Mais la voie d'administration n'a pas montré toute différence entre les deux sites (*χ*^2^ = 0.78, *p* = 0.37).

**Conclusion:**

Les anomalies ont été trouvées dans les deux sites et couvrent les quatre domaines évalués (dans le dosage des médicaments, la durée de traitement, et dans le voie d'administration et les intervalles entre administrations des médicaments). Par conséquent, nous concluons que le succès dans la mise en oeuvre des lignes directrices ne peut être fondé que sur des preuves scientifiques, mais doit tenir compte de certains autres facteurs contextuels.

## Introduction

### Key focus

Malaria remains the number one killer in Africa. Despite training, provision of medical supplies, regular monitoring and evaluation of programmes, the quality of care remains substandard mainly in primary health care.

### Background

Malaria represents a global disease with almost 40% of the world population living in endemic areas of malaria.^1^ Malaria is one of the major causes of anaemia in children in sub-Saharan Africa, accounting for an estimated 18% of the disability-adjusted life years (DALY) lost because of anaemia.^1^ The number of deaths attributable to malaria associated with anaemia is estimated at 190 000–974 000 per year amongst children less than 5 years.^2^

Current figures show that people living below the poverty line are the most affected by malaria with 58% of malaria incidences occurring in the poorest 20% of the world's population. Unfortunately, these patients receive the worst care which in turn has catastrophic economic consequences. This social vulnerability requires better understanding for improving deployment, access, quality, and use of effective anti-malaria interventions.^3^

Vertical malaria control programmes failed in many countries during the 1960s and 1970s. This lead to a resolution adopted by the 38th World Health Assembly in 1985 recommending that malaria control activities be developed as an integral part of primary health care systems at the district level.^4^ Immediately after its implementation, weaknesses were noted in the monitoring and evaluation. In countries such as the Congo, this was exacerbated by an inadequate health system as a result of armed conflicts which resulted in a shift of attention from malaria control measures to other priorities.

As a response to this acute crisis, Roll Back Malaria Initiatives and other partners through the National Malaria Control Programme decided to alleviate the situation by carrying out additional activities and strengthening those already in place. These measures comprised improving the supply of drugs and medical equipment, ITN (insecticide-treated nets) distribution, as well as the supervision, monitoring and evaluation and strengthening of human resources in health care through training.^3^

Good practice is, however, not always the resultant of training, which sometimes produces certificate holders who are not committed leaders or implementers.^4^ Furthermore, one survey suggested that only 50% of training investments eventually yield individual organisational improvements^5^ and dissemination of information. Training also transfers discrete skills and techniques in participants rather than long-term efficacy.^6^ As a result we cannot presume high quality of care from health professionals after their training.

### Trends

The correct use of anti-malarial drugs is a requisite for therapeutic success and the control of the spread of drug resistance. Hence, resistance is more likely to emerge when background immunity is weak, in the presence of low transmission and intense drug pressure,^7^ a high parasite burden and a low level of drugs in the blood.^8^ Irrational prescription practices have shown their influence as well on the emergence of resistance to conventional anti-malarial drugs.^9^ Consequently, the success of a new treatment policy would depend on the adherence to treatment guidelines by health providers and patients.^10^

A determination of discrepancies between the malaria guidelines and the current practice seems paramount to assess weaknesses and to improve the treatment. New guidelines using artemisinin-based combination therapies have been adopted in the African countries since 2006 as a consequence of the high rate of resistance to traditional anti-malarial drugs.

There are various factors that should be kept in mind when a health system is implementing new guidelines, such as:

- the political climate and fluidity of national borders
- efficacy and effectiveness
- the health care system
- the cost
- treatment seeking practices
- replacement drug selection
- procurement and new drug introduction
- the system of drug distribution
- drug safety
- quality assurance
- lead-time to policy change and drug's use.^11^

In anticipation to prospective problems which could arise from those implementation requirements of the current artemisinin-based combination therapies (ACTs), we reasoned that lessons learned from the misuse of the previous malaria guidelines could assist in rationalising the current guidelines which are 20–40 times more expensive than the conventional anti-malarial medicines.^12^

As a further complication, evidence have shown that changing and implementing new drug policies is complex when the transition includes well-known drugs such as sulfadoxine and pyrimethamine that used to be commonly prescribed, were easy to administer, inexpensive, and readily available.^11, 13^

We hypothesise that lessons learned from this transition between sulfadoxine-pyrimethamine therapy first line treatment and the combination regimen in terms of the use of the anti-malarial, can be extrapolated to the new combination regime and thus reduce the likelihood of misuse.

Several studies on malaria in Congo have been conducted up to now, but most of these have focused either on evaluation of the prescription of anti-malaria drugs,^14^ evaluation of anti-malarial activities, and toxicities of plants in malaria therapy,^15^ or on epidemiological studies,^16^ to mention a few. There is a paucity of studies scrutinising malaria patient management.

At the time when the study was carried out, there was, despite the introduction of ACTs, overlapping use of sulfadoxine-pyrimethamine and artemisinin combination therapies (amodiaquine-arthemeter) as first line options in uncomplicated malaria. In the event of complicated malaria, quinine injection was used in peripheral facilities (intramuscular or intrarectal) before referral to a high level in the health system. Parenteral quinine was used as a loading dose, followed by oral quinine during the maintenance period. However, few patients were still taking chloroquine for 3 days (abandoned in 2001 in Congo in favour of sulfadoxine-pyrimethamine).

### Objectives

We therefore conduct this study aimed at assessing the use of anti-malarial drugs in case management in primary health care in Goma health district with objectives to assess the quality of management in two health centres and to compare this quality between a suburban and an urban health centres in Goma health zone, Congo.

### Contribution to field

This study will provide evidence of potential misuse of anti-malarial medication in primary health care in terms of dosage, duration of treatment, routes and intervals of administration, which will be used to improve the management, the quality of care and which will delay the onset of resistance to current anti-malarial drugs (arthemeter combination therapy).

This study will contribute to the improvement of the standard of malaria case management as a proxy of determining the points of weakness in the prescription, administration and monitoring of malaria treatment at the first level of care. Indirectly, it will prevent toxicity or substandard care in individual patients, minimise resurgence of resistance, reduce the cost of care and increase the well-being of prospective malaria patients (cost-effective care) in the long run.

## Ethical considerations

Authorisation to conduct the study was sought from and granted by the ‘Médecin chef de zone de Santé de Goma’ (District Health Officer of Goma) and the management team of the two facilities. The benefits of this study outweighed the risks to participants when improvement in the management of common diseases such as malaria, was taken into consideration.

Furthermore, data collected for this study were stored on a computer with very limited access to outsiders to the study. With the removal of all identifiers, it was highly improbable for any reader to establish any link between the research data and participants. This guaranteed the anonymity of participants. The report has been written in an objective way to reflect the situation researched as well as to minimise risk to participants throughout the study in order to increase the credibility of the audit and ensure the protection of the participants. Ethical considerations such as consent, privacy, and freedom from harm, and the value-free nature that an audit shares with research could oblige an ethical approval beforehand.^17, 18^ However, for reasons stated above and given the absence of an ethics review board in our province and in the neighbourhood during the post-conflict era, we sought approval only from an institutional ethics review board of the Doctors on Call for Service (DOCS) Learning Centre; now a teaching hospital for Family Medicine residents.

## Methods

### Materials

The population of this study comprised all patients attended to during the period of study mentioned earlier.

### Setting

The study was carried out at two sites in Goma City in the Democratic Republic of the Congo. The *CCLK Health Centre*, located downtown, is a church-owned health centre mainly used by poor, displaced communities in the south of Goma on the road to South Kivu Province. It is situated approximately 20 km from the city, and inhabited by internally displaced, poor people. It is a theological training site with a health centre used as a centre for training in community-oriented primary care and community health for residents studying for their Master's Degree in Family Medicine.

The second site, the *Casop Health Centre*, is a state-owned clinic which is located in Goma city-centre and serves mostly urban people in the lowest socio-economic group. It is located in a densely populated part of the city at the border between Congo and the Republic of Rwanda.

These two clinics were integrated in the district health system as two of the affiliated health centres around the Goma health district. Both centres meet the requirements for adequate staffing levels and have, apart from the mobile care, waiting rooms as well to observe patients for stabilisation and/or referral to district hospitals. The two centres have the same climatic conditions and socio economic status even though CASOP has lower standards of hygiene. Both centres are located in high endemic zones of malaria. The two centres draw on a user's fee system for cost recovery but part of the expenses are covered by an Irish NGO (Non-government organisation), Association Régionale d'Approvisionnement en Médicaments Essentiels [*Regional Association for the supply of essential drugs*], ASTRAMES, for common ailments, including malaria. The management of malaria is in line with the National Malaria Control Program of which the guidelines are reported in Box 1.

### Design

It is a descriptive retrospective study.

### Data collection

One thousand two-hundred-and-seventy-two malaria patients were reviewed between January 2004 and April 2005.

Data were collected with regard to demographic (age, sex, address and date) information, and clinical (symptoms, treatment given, dosage, administration route, the duration of treatment, the route of administration, the dose recorded and the indication) information, by using the malaria register's review.

### Definitions

For the purpose of the study:

- Under dosage (and over dosage) respectively refer to the administration of a single dose of less (or more) than 25 mg base/[kg body weight], (1.25 mg base/[kg body weight]) sulfadoxine-pyrimethamine (SP) in simple cases, or less (or more) than 30 mg/[kg body weight] of quinine in severe cases. For artemisine-based combination therapy in uncomplicated cases, artesunate is administered at a higher dosage than 4 mg/[kg body weight], (and lower than 10 mg/[kg body weight]) of amodiaquine over a period of 3 days.
- Inappropriate Routes include any administration of medication in a route which is not in line with the severity of the malaria incidence. An example would be the administration of parenteral quinine in an uncomplicated case of malaria and vice versa.
- Long duration of treatment was any administration of drugs more than once for SP, 7 days for quinine, and 3 days of artemisine combinations therapies or chloroquine for a few patients.
- Inappropriate intervals were defined as the administration of quinine more or less than three times per day (or every 8 hours), or of ACT less or more than twice per day (the same applied in the case of quinine in infusion), and for chloroquine every 12 hours.

No documented data were classified as abnormal during analysis.

### Analysing

Quality was defined as the degree to which health services for individuals and populations increase the likelihood of desired health outcomes and are consistent with current knowledge^19^ in terms of dosage given, intervals between administration, route of administration, and duration of treatment.

Variables were assessed against the Congolese National Guidelines of Malaria programme; borrowed from WHO's guidelines (Box 1). The assessment was carried out firstly, comparing the reported medicine with the recommended regimen in the guidelines. Secondly, the appropriateness of the prescribed drugs in terms of dosage, duration, route and the intervals of administration, was assessed. Finally, the two sites were compared to identify possible variations across sites.

Data analysis was carried out using SPSS 11.0 for Windows, (SPSS Inc., Chicago, IL) software. Firstly, a descriptive statistical analysis was carried out. Proportions (and their 95% confidence intervals) were computed. Chi-square (with Yates's correction or Fischer's exact test when appropriate) was calculated to assess categorical variables. Significant differences were detected when *p* ≤ 0.05.

## Results

The majority of the participants, 806 (66.7%), were aged 5 years or older, and 654 (56.5%) participants were from within the same catchment area as the clinics (Table 1). The sex-ratio is almost equal to 1 and the two facilities have approximately the same proportion of participants (Table 1).

**TABLE 1:** Socio-demographic data of respondents.

Socio-demographic data	*n*	%
**Age (*n* = 1209)**		
≤ 4 years	403	33.30
≥ 5 years	806	66.70
**Location of patients (*n* = 1157)**		
In the catchment	654	56.50
Outside the catchment	422	33.50
Outside the district	165	14.30
Not recorded	27	2.30
**Health facility (*n* = 1269)**		
CCLK	626	49.30
CASOP	643	50.70
**Sex (*n* = 1270)**		
Male	644	50.70
Female	626	49.30

*Source*: Authors’ original data

CCLK, Centre Chrétien du Lac Kivu [*Lake Kivu Christian Centre*]; CASOP, Caisse de Solidarité Ouvrière et Paysanne [*Fund of Solidarity Workers and Peasants*].

*n*, given as means of number.

Under dosage is more prevalent in CCLK with 52 patients (62.5%; 95% CI, 52% – 71.8%), whilst over dosage is more common in CASOP with 64 patients (80%; 95% CI, 69.9% – 87.2%), (Table 2). The shortest duration of treatment is found mainly in CCLK with 15 patients (93.7%; 95% CI, 71.6% – 98.8%).

**TABLE 2:** Quality Insurance of malaria case management assessment.

Quality Insurance	Health facility						

	CCLK			CASOP			Total
		
	*n*	%	95% CI	*n*	%	95% CI	*N*
**Dosage (*n* = 586)**							
Accurate dosage	503	51.2	48.1–54.3	478	48.7	45.6–51.8	981
High dosage	16	20.0	12.7–30.0	64	80.0	69.9–87.2	80
Low dosage	55	62.5	52.0–71.8	33	37.5	28.1–47.9	88
Not documented	12	52.1	32.9–70.7	11	47.8	29.5–67.0	23
**Total**	**586**	-	-	**586**	-	-	**1172**
**Duration of R_x_ (*n* = 586)**							
Normal	560	49.2	46.3–52.1	577	50.7	47.8–53.6	1172
Short	15	93.7	71.6–98.8	1	6.2	1.1–28.3	16
Not documented	11	57.8	36.2–76.8	8	42.1	23.1–63.7	19
**Total**	**586**	-	-	**586**	-	-	**1172**
**Intervals (*n* = 587)**							
Appropriate	584	50.2	47.3–53	579	49.7	46.9–52.6	1163
Inappropriate	3	17.6	6.1–41.0	14	82.3	58.9–93.8	17
**Total**	**587**	-	-	**593**	-	-	**1180**
**Routes (*n* = 620)**							
Intravenous	61	37.4	30.3–45.0	102	62.5	54.9–69.6	163
Oral	515	51.5	48.4–54.6	484	48.4	45.3–51.5	999
Intra rectal	1	-	-	0	0	0	1
Not recorded	43	43	33.7–52.7	57	57	47.2–66.2	100
**Total**	**620**	-	-	**643**	-	-	**1263**

*Source*: Authors’ original data

CCLK, Centre Chrétien du Lac Kivu [*Lake Kivu Christian Centre*]; CASOP, Caisse de Solidarité Ouvrière et Paysanne [*Fund of Solidarity Workers and Peasants*].

*n*, given as means of number; *N*, total number of participants; CI, confidence interval; R*_x_*, treatment.

CASOP had a high number of participants with inappropriate intervals between administrations, that is, 14 patients (82.3%, 95% CI 58.9% – 93%). Despite the low standard of the health care system, intravenous drugs were administered to 61 patients (37.4%; 95% CI, 30.3% – 45%) in CCLK against 102 patients (62.5%; 95% CI, 54.9% – 69.6%) in the CASOP health centre.

There was a statistically significant difference in the intervals of administration (*χ*^2^ = 7.11, *p* = 0.007), in durations of treatment (*χ*^2^ = 8.51, *p* = 0.003) and in dosages (*χ*^2^ = 3.91, *p* = 0.05) between the two health centres (Table 3). The routes of administration were used in the same manner at the two sites.

**TABLE 3:** Comparison of administrations between the two facilities.

Administration variables	CCLK		CASOP		Total	*χ*^2^	ddf	*p*
	
	*n*	%	*n*	%				
I**nterval**								
Normal	584	99.4	579	97.6	1117	7.11	1	0.007
Abnormal	3	0.5	14	2.4	-	-	-	-
**Duration**								
Normal	560	95.6	577	98.5	1172	8.51	1	0.003
Abnormal	26	4.4	9	1.5	-	-	-	-
**Dosage**								
Normal dosage	503	85.8	478	81.6	1172	3.91	1	0.05
Abnormal dosage	83	14.2	108	18.4	-	-	-	-
**Route**								
Abnormal route	61	10.6	102	9.2	1164	0.78	1	0.37
Normal route	515	89.4	1001	90.8	-	-	-	-

*Source*: Authors’ original data

CCLK, Centre Chrétien du Lac [*Lake Kivu Christian Centre*]; CASOP, Caisse de Solidarité Ouvrière et Paysanne [*Fund of Solidarity Workers and Peasants*].

*n*, given as means of number; ddf, denominator degrees of freedom; *p*-value, probability value; *χ*^2^, Chi-square.

Sulfadoxine and pyrimethamine were the drugs most used by 919 patients (78.3%; 95% CI, 74.9% – 81.3%) in CCLK, compared to 429 patients (66.7%; 95% CI, 63.0% – 70.3%) in CASOP, followed by quinine in 86 patients (13.7%; 95% CI, 11.3% – 16.7%) in CCLK, compared to 122 patients (16.4%; 95% CI, 16.1% – 22.2%) in CASOP (Table 4).

**TABLE 4:** Anti-malarial drugs used during the study period.

Anti-malarial drugs	Health facility					

	CCLK			CASOP		
	
	*n*	%	95% CI	*n*	%	95% CI
SP	490	78.3	74.9–81.3	429	66.7	63.0–70.3
Chloroquine	15	2.4	1.5–3.9	34	3.9	3.8–7.3
Quinine	86	13.7	11.3–16.7	122	16.4	16.1–22.2
ACT	35	5.6	4.0–7.7	58	7.3	7.0–11.5

**Total**	626	-	-	643	-	-

*Source*: Authors’ original data

Quinine was administered *per os*, intravenous and intramuscular.

SP and chloroquine were administered *per os*.

CCLK, Centre Chrétien du Lac Kivu [*Lake Kivu Christian Centre*]; CASOP, Caisse de Solidarité Ouvrière et Paysanne [*Fund of Solidarity Workers and Peasants*].

*n*, given as means of number; SP, sulfadoxine-pyrimethamine; ACT, artemisinin combination therapy; CI, confidence interval.

## Discussion

### Outline of the results

Irrational prescription practices such as the use of subtherapeutic doses and inappropriate administration frequencies were found in both sites in different proportions, confirming the sub-optimal treatment of malaria. Despite a high percentage of those abiding to guidelines, the frequency of the disease and its life threatening prognosis when mismanaged, cannot be overemphasised. A study carried out some years ago in Congo, for example, showed that the development of resistance to anti-malarial drugs in children lead to a 4.5-fold increase in the number of anaemic patients. We know that anaemia as a complication of malaria is the leading cause of death in paediatric age groups which constituted almost one-third of our participants in this study.

Mismanagement of malaria is not specific to our settings. A review carried out by Durrheim et al.^20^ found a failure in the administration of the correct anti-malarial drug, at the correct dosage and frequency amongst providers.

The explanation for this must be seated firstly in the high medicines’ unordered dosage in the register which was considered as an abnormality, nurses with no experience in the health centre's workload, the lack of in-service training targeting nurses who are in touch directly with patients instead of the staff in charge; and insufficient dissemination sessions of new guidelines due to financial constraints. The same explanation is applicable to the difference of intervals in administrations. However, the pronounced abnormal interval in administration in CASOP could reflect the higher lack of abidance to medical advices found in towns and cities, compared to suburban and rural settings. Furthermore, the busy daily schedule of coping strategies to face the high cost of living in the CASOP catchment area and surroundings can result in forgetfulness with regard to taking medication. The last postulation could explain also the higher use of intravenous quinine at the peripheral level. Seeking a quick relief and a return to normal life, patients prefer perfusion which can provide rapid relief and allow them to return to normal activities. From the perspective of the providers, in a system of self-funding and user-fees for services as mode of payment, intravenous quinine is almost 10 times more expensive than ACT. Some providers face a dilemma between the quality of care they have to preserve, and the resources they have to obtain from patients to sustain the facility on the other hand.

Patients with severe malaria in health centres should be provided pre-referral treatment of intramuscular quinine and should be transferred for full parenteral treatment and supportive care.^21, 22^ Paradoxically there was a high number of patients and a delay before referral, in the two facilities in the cases of severe malaria where quinine was administered intramuscularly in emergency situations.

This high rate use could be placed in the socio-economical context of Goma in general and CCLK-CASOP catchments areas in particular. The cost of parenteral quinine is several times higher than that of the arthemeter combination or SP and will enable a health centre to earn money quickly. Government-related funds were almost absent during and some years before this study, and consequently the misuse of quinine was aimed probably at the alleviation of the financial constraints that the health centres were experiencing. The facilities could then cope more easily with their monthly expenditure including utilities, salaries and other expenses.

Anecdotal reports acknowledge that fast symptom alleviation of parenteral quinine in conjunction with its relatively mild side effects encouraged patients to opt for quinine, even in uncomplicated incidences of malaria. The rapid onset of action of quinine causes patients to assume that they are cured and consequently they do not see any reason for a maintenance dosage, which results in incomplete treatment. In addition, quinine, well known by both patients and providers as an established medicine, manufactured in the country, and more competitive in terms of prices compared to ACT combination, remains the preferred medication.

This misuse of quinine in uncomplicated incidences of malaria is very common, irrespective of the level of care in Congo. The misuse is probably related to the national policy in drug management, the continuous use of some old medicines such as chloroquine despite the change to new drugs, and the difficult access to care at policy level.

Short course and inappropriate routes of administration are in addition to intervals of treatment, reasons non-specific to this study. These conditions are not found only in those two facilities.

It has been found, for example, that up to 84.1% of patients with uncomplicated malaria were prescribed quinine in Kinshasa in the casualty department of a teaching hospital in Kinshasa, Congo.^22^ Another study^24^ carried out on the prescription of quinine in several facilities, found under dosage and over dosage in respectively 66.7% and 2.7% of patients. Some prohibited routes of administration of quinine such as intramuscular administration, were used in 13.4% of patients throughout the course of treatment. Those weaknesses in anti-malaria drug use are very dangerous, because medication errors and adverse drug reactions (ADRs) may result in disability and even death.^25^

Concomitant use of new ACTs combination with chloroquine and sulfadoxine-pyrimethamine is a matter of great concern. Studies^26^ carried out previously have already demonstrated the emergence of some resistance to the two drugs; mostly in the eastern Congo where our study has been conducted. This will cause numerous preventable deaths, in addition to those due directly or indirectly to war and natural disasters in this fragile part of the country.

### Limitations

Some limitations of this research must be taken into account when interpreting the findings.

Providers’ self-report or interviews about their own performance have certain pitfalls which can lead to bias. Most of the time they provide information on what providers know and not necessarily what they routinely do.^27^ Furthermore, self-reported performance through some interviews is likely to overestimate what providers do, both for tasks they do not perform often, as well as for tasks they perform often. By conducting the register review we reduced the subjectivity of self-reported performance as stated above.

Secondly, the two sites have been purposefully selected; hence results of the current research cannot be extrapolated to other facilities within the district or beyond. However, with a detailed description of the study settings and the review of literature supporting our findings, we think that the results can be translated at least to similar settings.

Thirdly, by collecting data from registers, there is a risk that some officers can report what is ideal instead of writing up what really happens to minimise criticism from supervisors and employers. In future, this bias will be reduced by carrying out a prospective study and/or study with a triangulation of data using several approaches and data sources of information.

## Conclusion

This study highlighted weaknesses in malaria case management in terms of dosage, duration of treatment, intervals between doses and route of administration which present variations across the study's sites. Use of quinine in non-indicated patients remains also a reason of concern. All of this is taking place in a context of socio-economic constraints despite training in malaria case management. From the current study, we can conclude that use of guidelines in primary health care cannot only be tailored to scientific evidence for its success; in addition it must also consider social and economic contextual factors, as well as cultural and political contextual factors.

BOX 1 Adapted Summary of DRC National protocol of malaria treatment at PHC level.

First line treatment of malaria in non-severe malaria:

- Quinine is used in failure treatment after sulfadoxine-pyrimethamine (SP)
- Severe malaria
- Pregnant women
- Children under 3 months
- SP is prohibited during the last 3 and first 3 months of pregnancy
- SP is contraindicated for children less than 3 years old.

**Table:** Guidelines for the use of sulfadoxine-pyrimethamine (SP) in non-severe malaria as a factor of body weight and age:

Age group	Weight	*n* (520 mg)
	kg	tablets
**Months**		
3–11	5–10	0.50
**Years**		
1–2	11–13	0.75
3–5	14–20	1.00
6–9	21–30	1.50
10–12	31–40	2.00
13–18	41–60	2.50
**Adults**		
-	> 60	3.00

*Source*: Ministere de la santé DRC [*Ministry of Health, DRC*] (2005)^27^

*n* case of treatment failure with SP, quinine salt 10mg tid during 7 days.

*n*, given as means of number of tablets.

**Table:** Patients with severe malaria (intermediate level of care):

**Infusion of quinine**	
Loading dose	20 mg of quinine per kg in 5 mL – 10 mL of dextrose, 5% per kg, for 4 hours followed by 8 hours of rest
Maintenance dose	12 hours after starting the first dose: 10 mg of quinine per kg in 5–10 mL of dextrose 5% per kg of body weight for 4 hours. Repeat every 12 hours until the patient is be able to ingest by mouth. If the patient had taken quinine or mefloquine during the prior 7 days, the loading dose cannot be taken, and there should be proceeded directly with the maintenance dose.
**For pregnant women**	
16th week	After active foetus movements, give 3 tablets of SP of 525 mg stat.
28th week	Repeat the dose.
HIV and AIDS	The SP must be given three times, the last dose at the 32nd week of pregnancy.

*Source*: Ministere de la santé DRC [*Ministry of Health, DRC*] (2005)^27^

**Table:** Dosing schedule for artesunate + amodiaquine:

Age group	Dose in mg (number of tablets)					
	Artesunate (50 mg)			Amodiaquine (153 mg)		
	Day 1	Day 2	Day 3	Day 1	Day 2	Day 3
**Months**						
5–11	25 (0.5)	25	25	76 (1.0)	76	76
1–66	50 (1.0)	50	50	153 (1.0)	153	153
**Years**						
7–13	100 (2.0)	100	100	306 (2.0)	306	306
>13	200 (4.0)	200	200	612 (4.0)	612	612

*Source*: WHO 2006 ^28^

Note: The total recommended treatment is 4 mg/[kg body weight] of artesunate and 10 mg base/[kg body weight] of amodiaquine administered once a day for 3 days.
